# Multi-Costimulatory Pathways Drive the Antagonistic Pseudoalteromonas piscicida against the Dominant Pathogenic Vibrio harveyi in Mariculture: Insights from Proteomics and Metabolomics

**DOI:** 10.1128/spectrum.02444-22

**Published:** 2022-10-27

**Authors:** Wei Ren, Bingqing Xue, Feifei Cao, Hao Long, Yanhua Zeng, Xiang Zhang, Xiaoni Cai, Aiyou Huang, Zhenyu Xie

**Affiliations:** a State Key Laboratory of Marine Resource Utilization in the South China Sea, Hainan University, Haikou, Hainan, China; b Hainan Provincial Key Laboratory for Tropical Hydrobiology and Biotechnology, Hainan University, Haikou, Hainan, China; c College of Marine Sciences, Hainan University, Haikou, Hainan, China; d Laboratory of Development and Utilization of Marine Microbial Resource, Hainan University, Haikou, Hainan, China; University of Prince Edward Island

**Keywords:** pathogenic *Vibrio harveyi*, antagonistic bacteria, immune response, proteomic combined metabolomic analysis, costimulatory pathway, recirculating-mariculture system

## Abstract

Vibrio harveyi is the dominant pathogen in mariculture, and biocontrol of this pathogen using antagonistic probiotics is a long-standing biological challenge. Here, Pseudoalteromonas piscicida WCPW15003 as a probiotic effectively antagonized dominant pathogenic V. harveyi in a mariculture, with a growth-of-inhibition ratio of 6.3 h^−1^. The antagonistic activities of cells and intracellular components of WCPW15003 made a greater contribution to the antagonistic process than did extracellular metabolites and caused the dominance of WCPW15003 during the antagonistic process *in vitro*. WCPW15003 was safe for the pearl gentian grouper (♀ *Epinephelus fuscoguttatus* × ♂ *Epinephelus lanceolatus*) and, as a consequence of the antagonistic effect on V. harveyi, protected the fish from an immune response *in vivo*. A comprehensive combined proteomics and metabolomics analysis of antagonistic WCPW15003 and pathogenic V. harveyi in a coculture compared to a monoculture was performed to investigate the antagonistic molecular mechanisms. The results showed that during the antagonistic process, WCPW15003 in a coculture had significantly downregulated metabolic pathways for histidine metabolism, arginine biosynthesis, and phenylalanine metabolism, and upregulated glycerophospholipid metabolism, leading to a competitive advantage against the co-occurring species, V. harveyi. This defined a mechanism by which multi-costimulatory pathways drove *P. piscicida* WCPW15003 against V. harveyi.

**IMPORTANCE** V. harveyi as a dominant pathogen has become a major hazard in mariculture development and seafood safety, and biocontrol of this pathogen using antagonistic probiotic agents is a long-standing biological challenge. *P. piscicida* WCPW15003 has promise as a novel, safe, and effective bioagent for specifically inhibiting dominant pathogenic V. harveyi and protects mariculture animals from infection by this pathogen by moderating the host immune response, which is heavily driven by multi-costimulatory pathways in a coculture of WCPW15003 and V. harveyi. This work identified a direction for comprehensively elucidating the molecular mechanism of WCPW15003 antagonism against the dominant pathogen in mariculture using modern molecular biology techniques and provided deep insights into the advantages and potential of this antagonistic probiotic against V. harveyi for the construction of an environmentally friendly, recirculating mariculture system.

## INTRODUCTION

*Vibrio* is a major bacterial disease in aquatic ecosystems and can cause serious infection in almost all aquatic animals, including penaeid shrimp, sea cucumber, and various fish species (1). *Vibrio* is becoming one of the most challenging hazards to the development of mariculture and safety of seafood as a result of the frequent outbreaks of epidemic diseases. Of all *Vibrio* species, V. harveyi has been identified as a dominant pathogen which causes scale drop, muscle necrosis, and acute mortality of commercial animals, leading to serious economic mariculture losses (2, 3). *Vibrio* as a cause of aquatic animal disease was first recorded in the 1990s (4); subsequently, disease outbreaks caused by this bacterium have been reported in multiple regions, such as India, China, southeast Asia, southern Europe, South America, and the Mediterranean Sea (5, 6). In the process of commercial aquaculture, extensive prophylactic and therapeutic antibiotics have been used as inhibitors of pathogenic V. harveyi to blindly pursue high yield and income, triggering the development of antimicrobial resistance (7–9).

An inhibitor does not need to be an antibiotic and may instead be involved in bacterium-bacterium communication by regulating metabolism or physiology such as quorum sensing, thereby driving changes in the bacterial community structure (10, 11). Additionally, with increased awareness of the quality and safety of aquatic products, the rising concern of antibiotic-resistant pathogens has led to the emergence of alternative disease prevention strategies, such as the application of nonpathogenic bacteria with antagonistic properties as potential alternative biocontrol agents (7, 12, 13). Some antagonistic bacteria against V. harveyi, such as Pseudomonas spp. (14–16), *Lactobacillus* spp. (17, 18), *Pediococcus* spp. (19, 20), and *Bacillus* spp. (21), offer promising alternatives to chemicals and antibiotics in mariculture due to their capacity to produce inhibitory compounds without impacting the overall biochemical balance of the water environment. However, most research in this field has focused only on the physiological antagonistic properties of bacteria and their inhibitory compounds against V. harveyi, not on safe and efficient vaccines as bio-antagonistic agents for controlling it. Moreover, knowledge of antagonistic mechanisms in coculture or natural mariculture is still very limited.

The natural environment is an ecosystem with numerous coexisting bacterial species, in which biotic antagonism is an undoubtedly complex process. Thus, studies on the activity, metabolism, and interactions of antagonistic bacteria against pathogenic bacteria in coculture are essential, because both types of bacteria may exhibit antagonistic interactions through regulating metabolisms and enzyme activities (22). Furthermore, bacterial-bacterial antagonism is reported as the main factor involved in bacteria-mediated carbon cycling in natural ecosystems (23, 24).

Previously, we isolated a novel marine antagonistic bacterium from Hainan Island, China, Pseudoalteromonas piscicida WCPW15003, which can effectively inhibit the dominant pathogen V. harveyi in mariculture water. In this work, we comprehensively characterized the antagonistic properties of *P. piscicida* against V. harveyi *in vitro* and *in vivo*, including antagonistic activity, safety, and fish immune response. Additionally, given the complexity of antagonistic process and interrelations, we performed a comprehensive multiomics analysis of antagonistic *P. piscicida* and pathogenic V. harveyi in a coculture versus monoculture to reveal the key metabolic pathways driving the antagonistic process in coculture. Our results provide novel insights for comprehensively elucidating this antagonistic mechanism in a more complex and natural environment for the construction of an environmentally friendly mariculture model.

## RESULTS

### Antagonistic activity of *P. piscicida* against *V. harveyi* in *vitro*.

**FIG 1:**
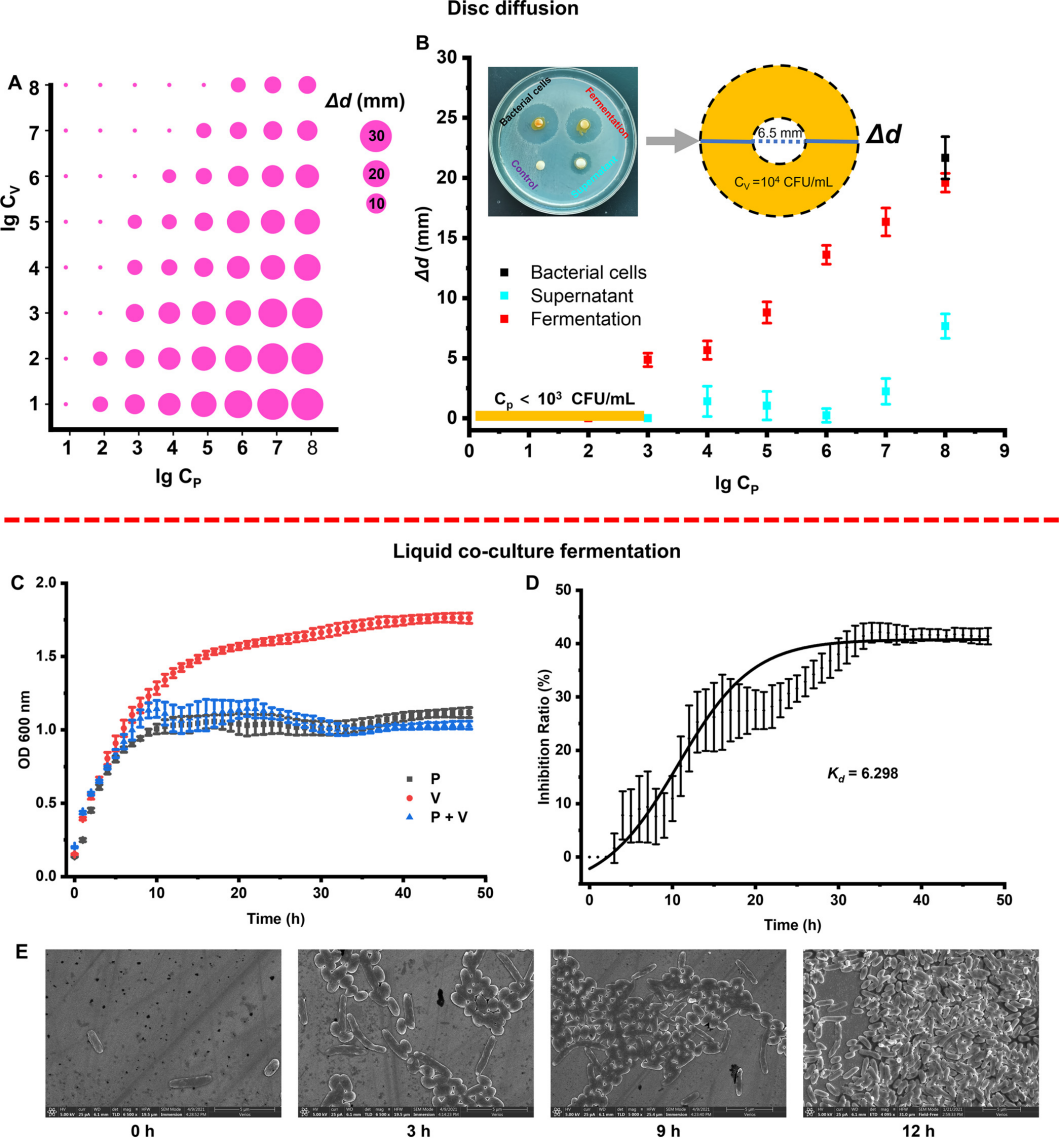
Antagonistic activity of Pseudoalteromonas piscicida against Vibrio harveyi in a disk-diffusion assay, and growth of *P. piscicida* and V. harveyi in liquid coculture. (A) Different initial concentrations of *P. piscicida* against different initial concentrations of V. harveyi in the disks. (B) Bacterial cells (without concentration gradient), cell-free supernatants, and fermented broth from different concentrations of *P. piscicida* against 10^4^ CFU/mL V. harveyi in the disk. (C) Growth curves of *P. piscicida* and V. harveyi in coculture and monoculture. (D) Growth curves (obtained by nonlinear fit) of the inhibition ratio of *P. piscicida* against V. harveyi, wherethe inhibition ratio is defined as the ratio of the difference between the OD values of V. harveyi (cultured individually) and the OD values of the two strains in coculture divided by the OD value of V. harveyi; (E) Electron microscopy examination of *P. piscicida* and V. harveyi in liquid coculture at different time periods (0 to 12 h).

A disk-diffusion experiment showed that the antagonistic activity of *P. piscicida* WCPW15003 against a certain concentration (10 to 10^8^ CFU/mL) of V. harveyi gradually increased as the initial concentration of *P. piscicida* increased, while activity gradually decreased as the concentration of V. harveyi increased (Fig. 1A). *P. piscicida* did not display significant antagonistic activity when the bacterial concentration was less than 10^3^ CFU/mL. When the concentration of *P. piscicida* (fermented broth) was higher than 10^3^ CFU/mL, the inhibitory activity continued to increase (Fig. 1B). As shown in Fig. 1B, the highest level of control of V. harveyi at 10^4^ CFU/mL on the pathogen plate was obtained with 10^8^ CFU bacterial cells of *P. piscicida*, which effectively inhibited the pathogen’s growth with slightly less inhibitory activity by the fermented broth; whereas the supernatant of *P. piscicida* exhibited only weak antagonistic activity, even at a high concentration, indicating that the antagonistic activity of cells or intracellular components of *P. piscicida* made a greater contribution to the antagonistic process than did extracellular metabolites (centrifuged supernatant; Fig. 1B).

The growth of *P. piscicida* and V. harveyi in coculture and monoculture was measured and assessed through liquid fermentation (Fig. 1C to E). As shown in Fig. 1C, the growth of the liquid coculture was consistent with that of *P. piscicida* cultured individually, and the antagonistic effect on V. harveyi increased throughout the fermentation. The inhibition ratio was regarded as an S-type growth curve described by the first-order rate equation *Y*_t_ = *Y*_0_ + a(1 – e^–kx^), where *Y*_t_ and *Y*_0_ are the inhibition ratios at a given culture time *t* and 0 h, respectively; *a* is the fitting coefficient; and *k* is the observed rate constant and the growth-of-inhibition ratio was approximately 6.3 h^−1^ (Fig. 1D). Bacterial morphology in the coculture was observed by scanning electron microscopy (SEM) (Fig. 1E), and the results showed that *P. piscicida* quantitatively had a dominance advantage with prolonged time compared with V. harveyi, which was entirely consistent with the liquid coculture results (Fig. 1C).

### Antagonistic activity of *P. piscicida* against *V. harveyi in vivo*.

**FIG 2:**
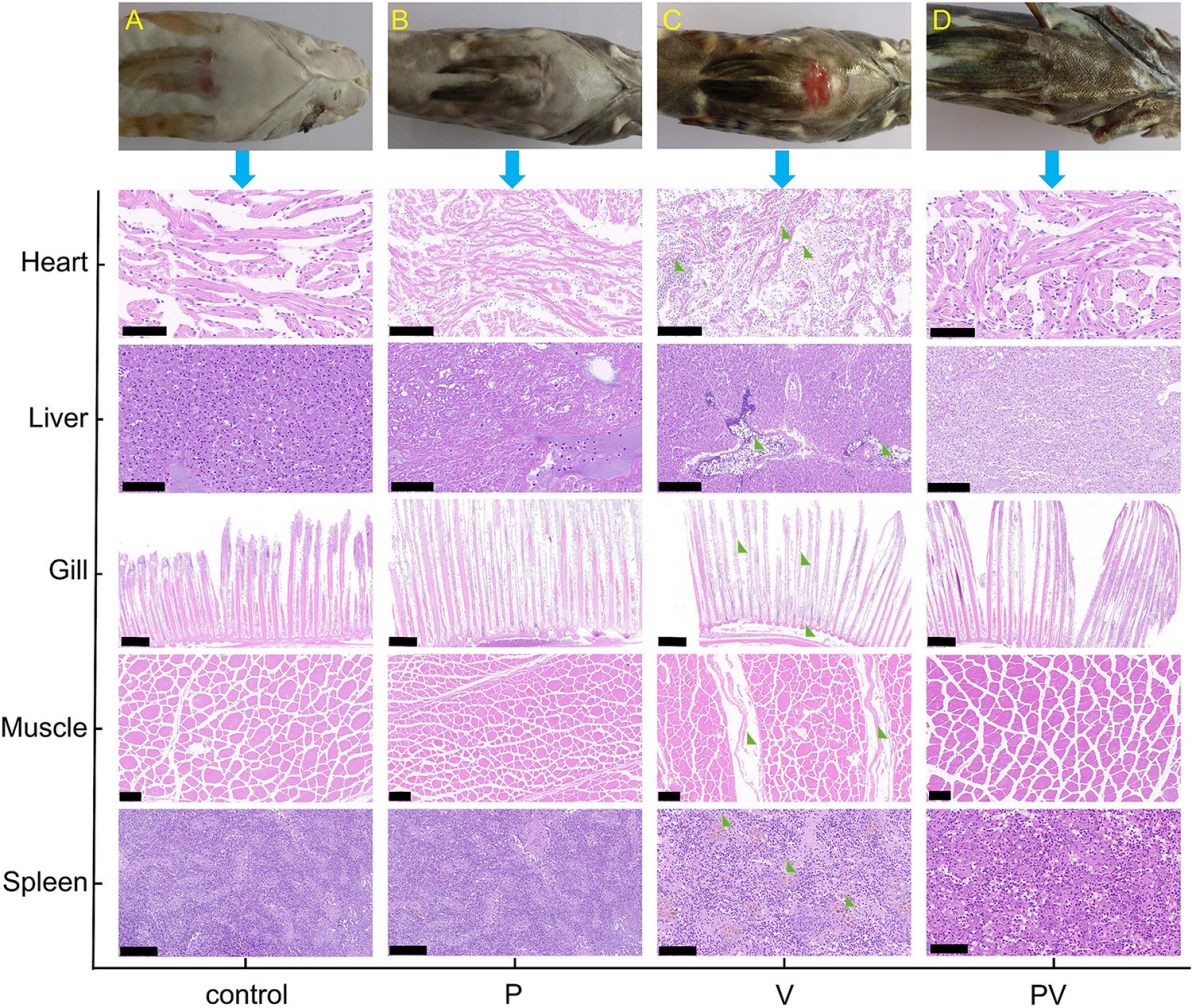
Photographs of fish bodies and histological changes in the heart, liver, gills, muscles, and spleen from four infected fish groups after rearing for 10 days. Control (A), *P. piscicida* monoculture (P group) (B), *V. harveyi* monoculture (V group) (C), and coculture of these two strains (PV group) (D). Green triangles indicate histological abnormalities. Scale bars: heart, 50 μm; liver, 50 μm; gill, 500 μm; muscle, 100 μm; spleen, 50 μm.

The antagonistic activity of *P. piscicida* against V. harveyi was evaluated *in vivo*. As shown in Fig. 2, compared with the control (Fig. 2A), *P. piscicida* in monoculture (P) (Fig. 2B), and *P. piscicida* and *V. harveyi* in coculture (PV) groups (Fig. 2D), the fish infected with V. harveyi (*V. harveyi* in monoculture [V] group; Fig. 2C) exhibited obvious symptoms of hemorrhage, septicemia, darkened skin, and ulcers on the skin surface, while fish in the PV group did not display similar symptoms except for the darkened skin. No obvious symptoms were detected in the control or P groups. The histological abnormalities of the V group were particularly noticeable compared with those of the control, P, and PV groups (results of histological examination shown in Fig. 2). The cardiac muscle fibers were disordered, partially dissolved, and torn; the liver cells were severely damaged and showed vacuolar changes and infiltration of inflammatory cells and neutrophils; the gill lamellas displayed cavities and were partially dissolved at the base and the gill filaments exhibited necrotic, uneven, and shortened fibers; muscle fibers were irregular, partially dissolved, and deformed with widened spaces; erythrocytes were increased in the spleen with a significant reddish color; and the spleens of fish infected with V. harveyi showed significantly more macrophages and a larger volume of melano-macrophage centers.

**FIG 3:**
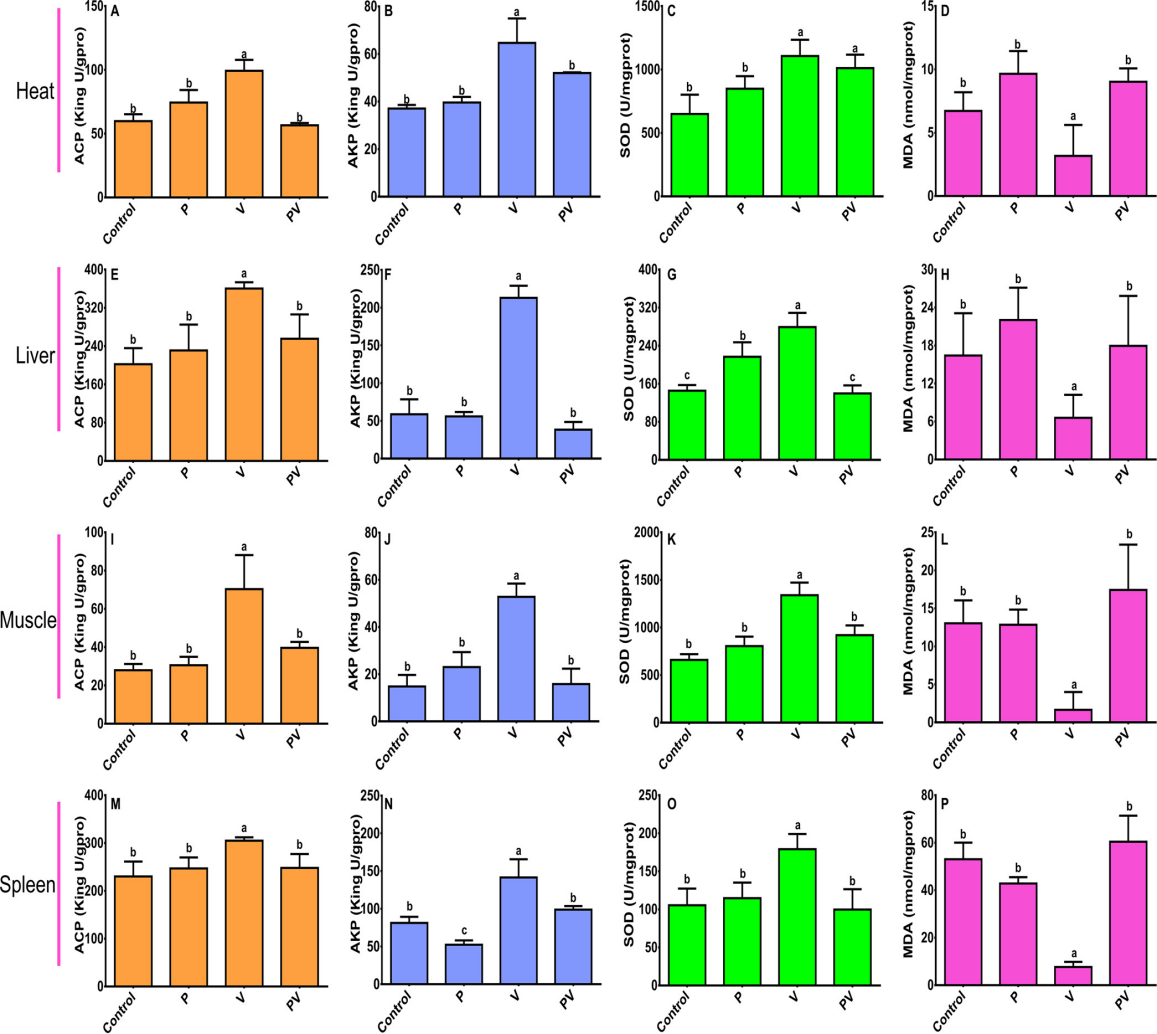
Immune-related enzymes in infected fish, including acid phosphatase (ACP), alkaline phosphatase (AKP), superoxide dismutase (SOD), and malondialdehyde (MDA), in hearts (A to D), livers (E to H), muscles (I to L), and spleens (M to P) of groupers from control, P, V, and PV groups after rearing for 10 days. Tissue samples of organs from each group were taken from three living fish randomly selected after rearing for 10 days.

The activity of the immune-related enzymes acid phosphatase (ACP), alkaline phosphatase (AKP), superoxide dismutase (SOD), and malondialdehyde (MDA) in the hearts, muscles, spleens, and livers of the fish from the control, P, V, and PV groups were analyzed after rearing for 10 days (Fig. 3). Interestedly, the detected immune-related enzymes displayed the same trend in different organs; specifically, the V group exhibited notably higher activities of ACP, AKP, and SOD compared to the P, PV, and control groups (*P* < 0.05) in each organ, as well as a remarkably lower level of MDA. A notably higher level of ACP activity was detected in the livers and spleens than in the hearts and muscles, while a notably higher level of SOD activity was detected in the hearts and muscles than in the livers and spleens. Although the V group exhibited notably higher activities of AKP in each organ, the livers of the V group displayed the highest AKP activity.

### Overview of metabolomic and proteomic variations during *P. piscicida* activity against *V. harveyi*.

**FIG 4:**
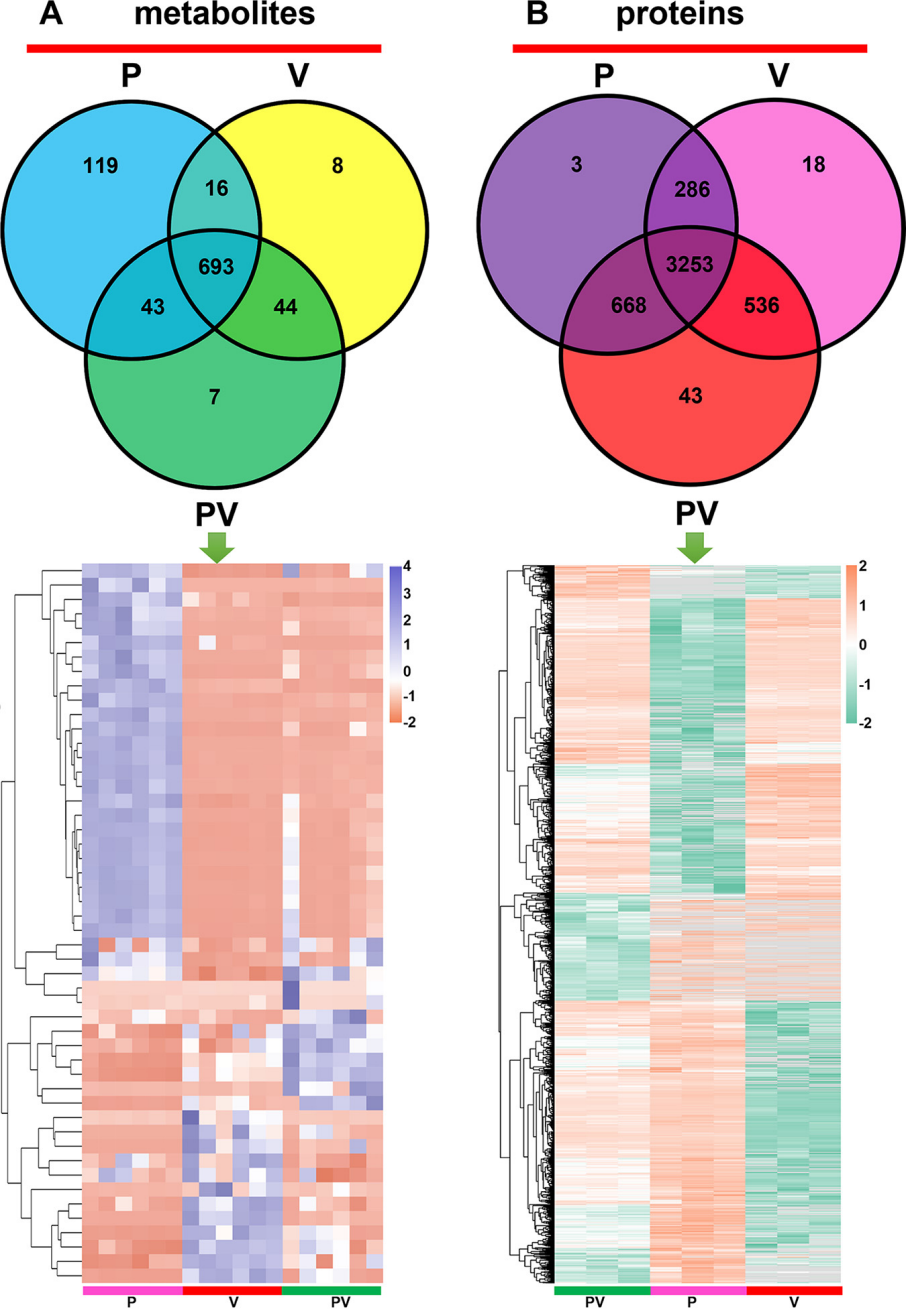
Overview of metabolomics (A) and proteomics (B) of *P. piscicida* (P group), V. harveyi (V group), and coculture of these two strains (PV group). P group: 871 metabolites and 4,210 proteins/V group: 761 metabolites and 4,093 proteins. PV group: 787 metabolites and 4,500 proteins.

The metabolomics and proteomics were explored to investigate global variations during the antagonistic process of *P. piscicida* against V. harveyi. A total of 930 metabolites and 4,807 proteins were detected in all three groups (Fig. 4; details of metabolomics and proteomics are shown in Tables S1 and S2, respectively), of which 276 metabolites and 2,238 proteins were annotated in KEGG. As shown in Fig. 4, the metabolites and proteins in each group were as follows: P group, 871 metabolites and 4,210 proteins; V group, 761 metabolites and 4,093 proteins; and PV group, 787 metabolites and 4,500 proteins. The highest abundances of metabolites and proteins were found in the P and PV groups, respectively. Most metabolites were detected in all three groups. The P group expressed 119 unique metabolites, whereas the V and PV groups harbored only eight and seven unique metabolites, respectively. Additionally, the expression of proteins exhibited similar results to that of the metabolomes, with 3,253 proteins shared by the three groups and only 3, 18, and 43 unique proteins found in the P, V, and PV groups, respectively.

### Differentially expressed metabolites and proteins during *P. piscicida* activity against *V. harveyi*.

**FIG 5:**
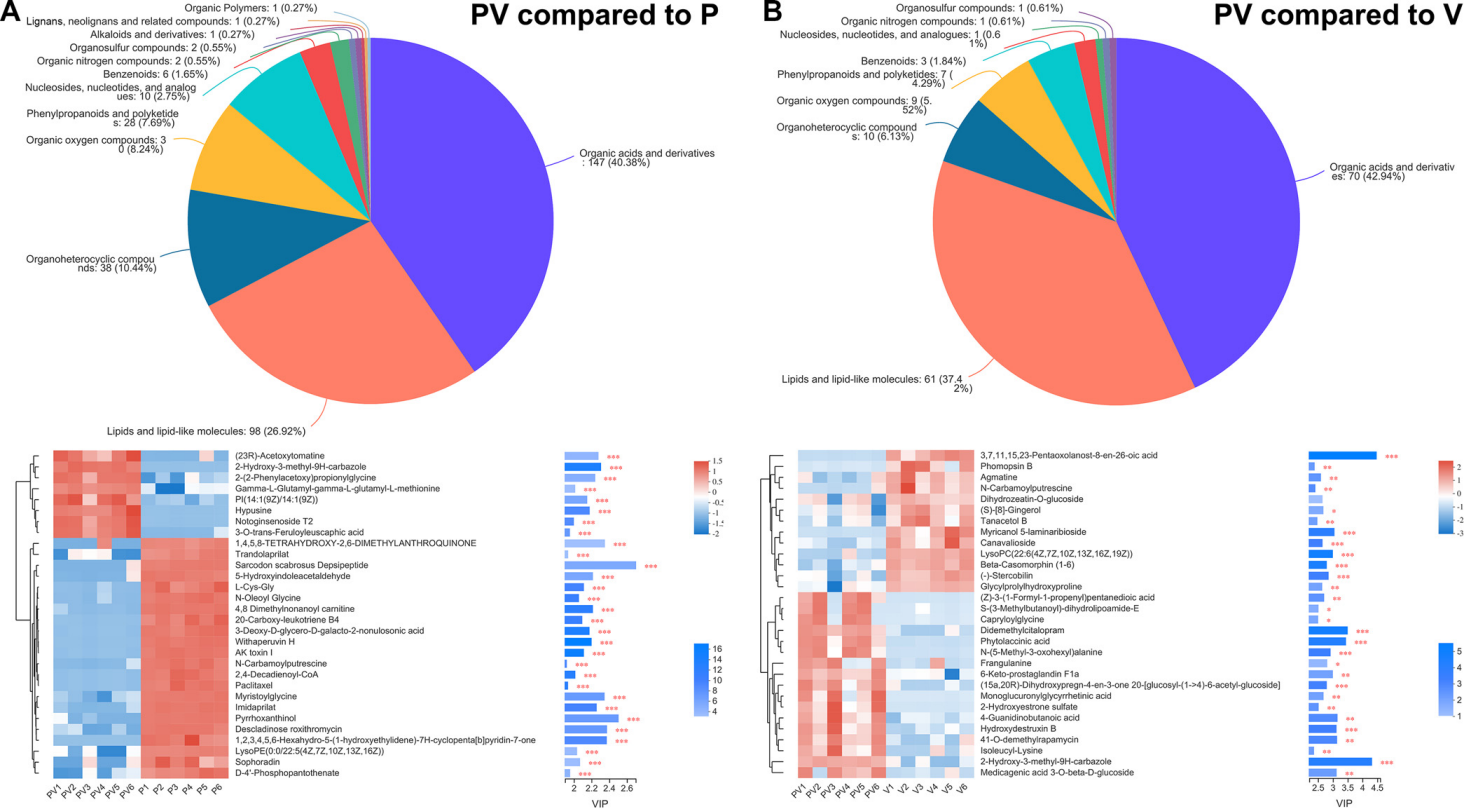
Comparison of the relative abundance of metabolites between the PV and P groups (A) and between the PV and V groups (B). Heat maps of the chemical composition in the PV group compared to the P and V groups to show changes in the top 30 metabolite concentrations. *, *P* < 0.05; **, *P* < 0.01; ***, *P* < 0.001.

The relative abundances of metabolites and proteins were compared between the PV and P groups and between the PV and P groups to investigate the differences between the coculture and monoculture of *P. piscicida* and V. harveyi. According to metabolomic identification using the Human Metabolome Database (HMDB), the classification of metabolites which accumulated at the highest frequency in the coculture (PV) group compared to the *P. piscicida* (P) and V. harveyi (V) groups are shown in Fig. 5. Comparing the PV and P groups (Fig. 5A), 454 metabolites exhibited relatively high concentrations, and these were divided into 12 categories: 147 organic acids/derivatives, 98 lipids/lipid-like molecules, 38 organoheterocyclic compounds, 30 organic oxygen compounds, 28 phenylpropanoids/polyketides, 10 nucleosides/nucleotides/analogues, 6 benzenoids, 2 organic nitrogen compounds, 2 organosulfur compounds, 1 alkaloid/derivative, 1 lignan/neolignan/related compound, and 1 organic polymer. Comparing the PV and V groups (Fig. 5B), 163 metabolites were divided into 10 categories: 70 organic acids/derivatives, 61 lipids/lipid-like molecules, 10 organoheterocyclic compounds, 9 organic oxygen compounds, 7 phenylpropanoids/polyketides, 3 benzenoids, 1 nucleoside/nucleotide/analogue, 1 organic nitrogen compound, and 1 organosulfur compound. Notably, there were more differential metabolites in the PV group compared to the P group than in the PV group compared to the V group, and the strains in coculture (PV group) clearly displayed more upregulated and downregulated differential metabolites than those in the monocultures. As shown in Fig. 5, the predominant accumulated chemical categories were organic acids/derivatives and lipids/lipid-like molecules, which are commonly regarded as significant metabolites of microbial growth (22).

**FIG 6:**
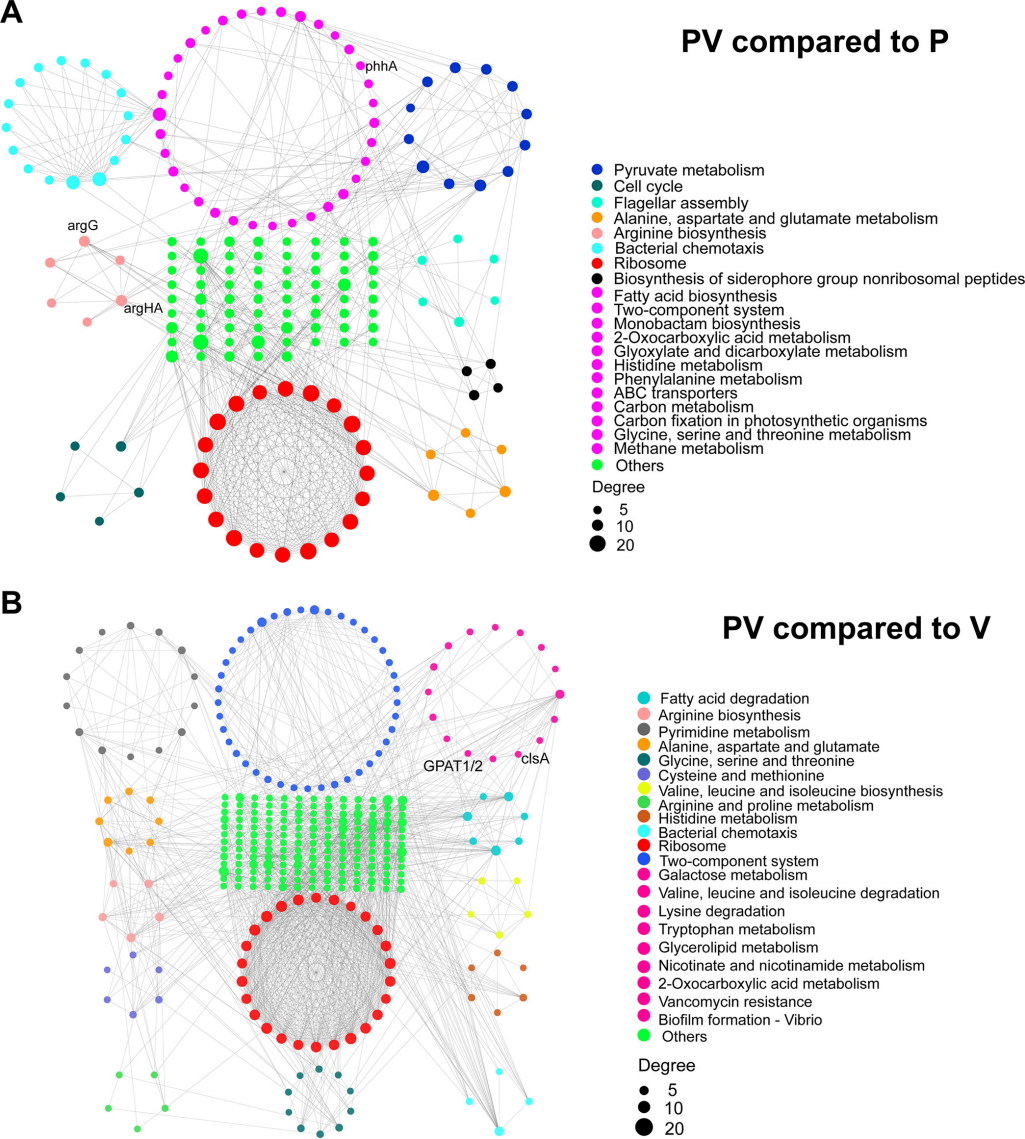
Analysis of protein-protein interaction networks in the PV compared to the P group (A) and the PV compared to theV group (B). Different colors identify various clusters according to KEGG pathway enrichment; *P < *0.05. Illustrations of protein-protein interaction networks with high confidence (0.07) were prepared using STRING v10.1 and visualized in Cytoscape v3.5.1.

To further understand the antagonistic effect of *P. piscicida* on V. harveyi, we evaluated metabolic pathway and protein-protein interaction (PPI) networks involving the observed enrichment of differentially expressed proteins, comparing the PV and P groups and the PV and V groups (Fig. 6). As noted by the PPIs (Fig. 6), the PV group was significantly enriched in 9 representative KEGG pathways compared to the P group (Fig. 6A): pyruvate metabolism, cell cycle, flagellar assembly, alanine/aspartate/glutamate metabolism, arginine biosynthesis, bacterial chemotaxis, ribosome, and biosynthesis of siderophore group nonribosomal peptides. Compared to the V group, the PV group was significantly enriched in 13 representative KEGG pathways (Fig. 6B): fatty acid degradation, arginine biosynthesis, pyrimidine metabolism, alanine/aspartate/glutamate metabolism, glycine/serine/threonine metabolism, cysteine/methionine metabolism, arginine/proline metabolism, histidine metabolism, bacterial chemotaxis, and two-component systems. As expected, the PPI analysis indicated that multiple metabolic pathways drove the antagonistic *P. piscicida* against the pathogenic V. harveyi in coculture.

We also screened the metabolites and proteins that displayed significant differences (*P* < 0.05) in concentration between the coculture and monoculture. According to this criterion, 67 metabolites and 247 proteins were identified as differentially expressed in the PV compared to the P group (Table S3), while 24 metabolites and 366 proteins were identified as differentially expressed in the PV compared to the V group (Table S4).

**FIG 7:**
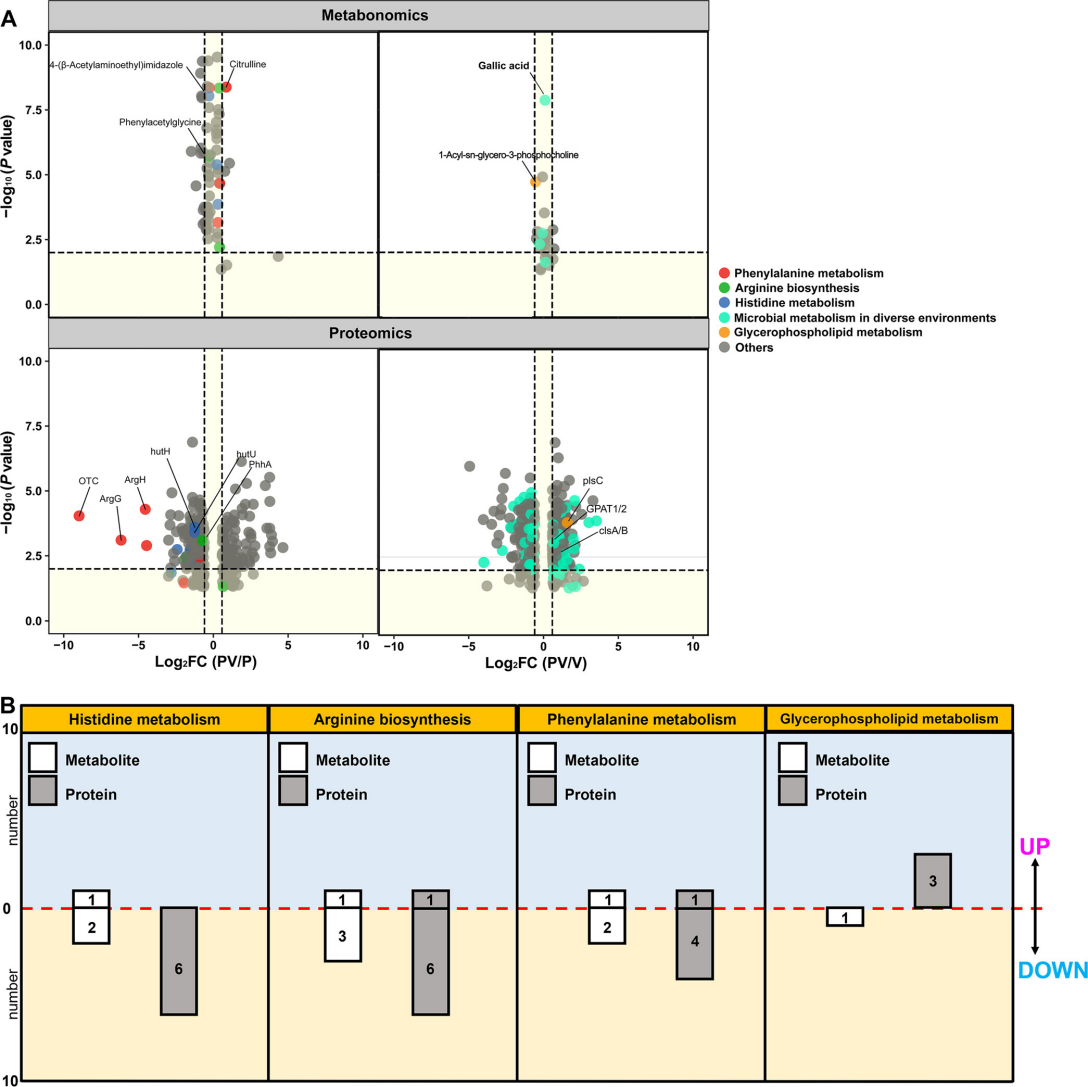
Changes in metabolite and protein abundance during the antagonistic process of *P. piscicida* against V. harveyi in coculture. (A) Volcano plot showing the differentially expressed metabolites and proteins (*P < *0.05) between coculture and monoculture, comparing the PV and P groups and the PV and V groups, and the preferential accumulation of metabolites and proteins assigned by KEGG to the major costimulatory pathways. (B) Significantly differentially expressed metabolites and proteins (*P < *0.05 and FC > 1.5) in the major costimulatory pathways: histidine metabolism, arginine biosynthesis, phenylalanine metabolism, and glycerophospholipid metabolism.

To better define how the metabolome and proteome were influenced by the coculture compared to that in the monoculture, we constructed volcano plots to compare levels of different metabolites and proteins in the coculture compared to the monoculture data sets based on both the log_2_ fold change (FC) and log_10_ *P* value for significance (Fig. 7A). Here, metabolites and proteins with *P* < 0.05 and FC > 1.5 were defined as significantly differentially expressed between groups. According to this criterion, when comparing the PV group to the P group, 20 metabolites and 247 proteins were identified as significantly differentially expressed, of which 5 metabolites and 110 proteins were upregulated, and 15 metabolites and 137 proteins were downregulated. When comparing the PV group to the V group, 3 metabolites and 366 proteins were identified as significantly differentially expressed, of which 3 metabolites and 192 proteins were upregulated and 174 proteins were downregulated (Fig. 7A).

Of the significant regulation of metabolites and proteins in the antagonistic process of *P. piscicida* against V. harveyi, the preferential accumulation of metabolites and proteins assigned by KEGG to the major costimulatory pathways was further investigated by a combined proteomic and metabolomic analysis. The results showed that metabolites and proteins in the costimulatory pathways of histidine metabolism, arginine biosynthesis, phenylalanine metabolism, and glycerophospholipid metabolism were significantly affected.

**FIG 8:**
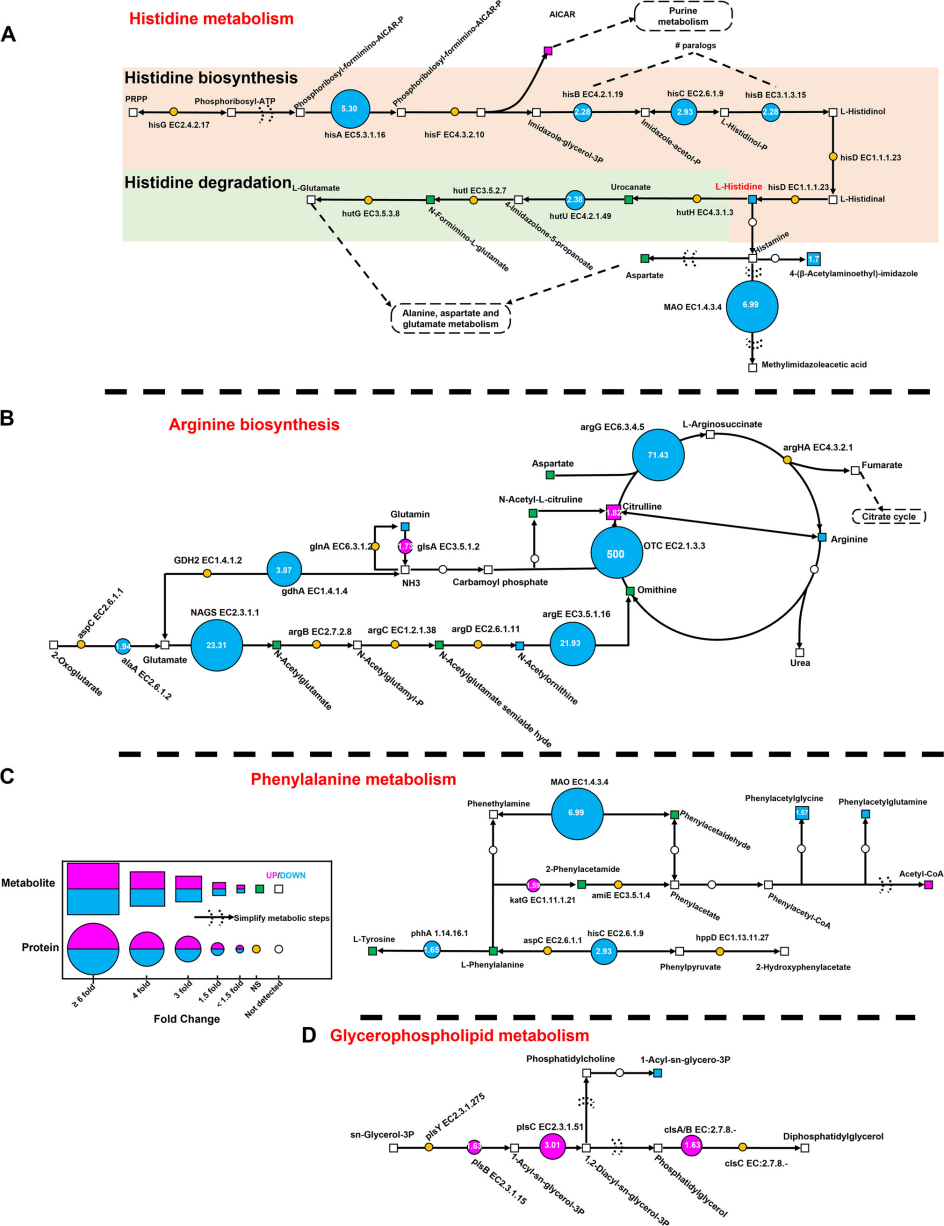
Simplified flow chart of the four major costimulatory pathways during the antagonistic process of *P. piscicida* against V. harveyi in coculture, describing changes in associated metabolites and relevant proteins and enzymes in coculture compared to monoculture: histidine metabolism (A), arginine biosynthesis (B), phenylalanine metabolism (C), and glycerophospholipid metabolism (D).

In histidine metabolism, one metabolite, 5′-phosphoribosyl-5-amino-4-imidazole carboxamide, was upregulated, while two metabolites and six proteins were downregulated (Fig. 7B). As shown in Fig. 8A, monoamine oxidase (MAO) was strongly downregulated (FC = 6.99), followed by phosphoribosylformimino-5-aminoimidazole carboxamide ribotide isomerase (HisA; FC = 5.3). Imidazoleglycerol-phosphate dehydratase (HisB), histidinol-phosphate aminotransferase (HisC), and urocanate hydratase (HutU) also displayed highly significant downregulation with FCs of >2. Additionally, 4-(β-acetylaminoethyl)-imidazole was significantly downregulated.

In arginine biosynthesis, citrulline and glutaminase (GlsA) were significantly upregulated with FCs of >1.5, while three metabolites and six proteins were significantly downregulated (Fig. 7B). As shown in Fig. 8B, ornithine carbamoyltransferase (OTC) was markedly downregulated (FC = 500), followed by argininosuccinate synthase (ArgG; FC = 71.43), *N*-acetylglutamate synthase (NAGS; FC = 23.31), and acetylornithine deacetylase (ArgE; FC = 21.93). Additionally, glutamate dehydrogenase (GlnA) and alanine transaminase (AlaA) both displayed significant downregulation with FCs of >1.9. Glutamine, *N*-acetylornithine, and arginine were also downregulated to varying degrees.

In phenylalanine metabolism, acetyl coenzyme A and catalase-peroxidase (KatG) were upregulated with FCs of >1.3, while two metabolites and three proteins were downregulated (Fig. 7B). As shown in Fig. 8C, MAO was strongly downregulated (FC = 6.99), followed by histidinol-phosphate aminotransferase (HisC; FC = 2.93) and phenylalanine-4-hydroxylase (PhhA; FC = 1.65). Additionally, phenylacetylglycine and phenylacetylglutamine also displayed significant downregulation with FCs > 1.3.

In glycerophospholipid metabolism (Fig. 7B and 8D), three proteins, 1-acyl-sn-glycerol-3-phosphate acyltransferase (PlsC; FC = 3.01), glycerol-3-phosphate O-acyltransferase (PlsB; FC = 1.63), and cardiolipin synthase (ClsA/B; FC = 1.63) were significantly upregulated with FCs of >1.5, while only one metabolite, 1-acyl-sn-glycero-3-phosphocholine, was downregulated (FC = 1.44).

Overall, histidine metabolism, arginine biosynthesis, and phenylalanine metabolism were significantly inhibited, whereas glycerophospholipid metabolism was activated during the antagonistic process of *P. piscicida* against V. harveyi in coculture.

## DISCUSSION

V. harveyi is the most harmful bacterial pathogen in mariculture, and infection in multiple host species is characterized by drug resistance and high toxicity (2). No effective or safe methods exist to prevent outbreaks of epidemics caused by this pathogen, which poses a great threat to aquatic food safety and causes direct economic losses to the mariculture industry. Although approaches for control of V. harveyi by increasing the use of prophylactics and therapeutics provide short-term curative effects, the frequent and excessive use of antibiotics will not only increase the drug resistance of the pathogenic strains but also accelerate the production of super-strains (7, 9), making antibiotics ineffective at controlling V. harveyi, destroying bacterial community structures in the aquaculture environment and in the aquatic animals themselves, and thus causing a series of issues in the ecosystem and reducing the quality of aquaculture products. Ecological prevention established by antagonistic probiotics is one of the most promising methods for the prevention and control of *Vibrio* pathogens. Reportedly, antagonistic probiotics against *Vibrio* pathogens, such as *Bacillus* spp., *Bifidobacterium* spp., and Pseudomonas spp., are characterized by unmatched advantages without disruption of the ecological balance or production of secondary pollution (25–27). Antagonistic probiotics display a high tolerance to harsh environments, such as high temperature, high pressure, and strong acidity or alkalinity, and they also produce a variety of antagonistic substances.

In recent years, although exploratory studies have been carried out on V. harveyi vaccines and antagonistic bacteria, most have focused on screening antagonistic strains of V. harveyi, and there is still a lack of safe and efficient vaccines and antagonistic agents that can effectively control this pathogen. More importantly, the antagonistic mechanism of V. harveyi has not been reported. In this work, an efficient antagonistic bacterium, *P. piscicida* WCPW15003, was successfully isolated from Hainan Island and its antagonistic peculiarity against V. harveyi was comprehensively analyzed, with insight into its antagonistic mechanisms elucidated through metabolomics and proteomics.

### *P. piscicida* effectively inhibited *V. harveyi in vitro*.

Multiple strains of Pseudomonas spp. have been reported to inhibit pathogenic bacteria, such as Vibrio anguillarum, Erwinia carotovora, *Alternaria canjani*, *Curvularia lunata*, Fusarium spp., *Bipolaris* spp., and *Helminthosporium* spp. (1). In our study, *P. piscicida* WCPW15003 as an antagonistic bacterium displayed excellent antagonistic activity against pathogenic V. harveyi with an S-type growth curve-like inhibition ratio *in vitro* and an increase in the inhibition ratio of up to 6.3 h^−1^ in coculture. We reason that the antagonistic interaction of *P. piscicida* against V. harveyi caused the dominance of this probiotic by competing with V. harveyi cells for nutrients; this was verified by SEM and also consistent with a coculture of *Pseudoalteromonas* sp. CF6-2 and Staphylococcus warneri MCCC1A00423 in artificial seawater that resulted in a strong reduction of the MCCC0423 strain with an increase of the CF6-2 strain over time (28).

Reportedly, the bacterium-bacterium antagonistic interaction is one of the main factors contributing to fluctuations in bacterial community structure (23). In this work, the antagonistic activity of cells or intracellular materials of *P. piscicida* made a greater contribution to the antagonistic process than did extracellular metabolites (Fig. 1B). Using microscopy, we closely observed *P. piscicida* and V. harveyi in coculture and found that one or a few V. harveyi cells were usually surrounded and attached to a number of *P. piscicida* cells (Fig. S2A in the supplemental material). A previous study found that the attached bacteria produced significantly greater inhibition compared to the free-living bacteria by producing antagonistic molecules (23). Moreover, it was clear that these two strains were linked through metabolite-like materials during the process of *P. piscicida* antagonizing V. harveyi (Fig. S2B). Therefore, we hypothesize that these links are channels for antagonistic material or energy communication and conversion, through which antagonistic functions were performed.

### *P. piscicida* effectively inhibited *V. harveyi in vivo*.

Vibriosis is a common problem in intensive mariculture with outbreaks at various culturing stages (29). V. harveyi is the main etiological agent of vibriosis, and aquatic animals infected with V. harveyi inevitably exhibit a series of lesions and symptoms without effective therapy, such as gastroenteritis, operculum nodules, scale exfoliation, muscle necrosis, skin ulcers, vasculitis, and tail rot disease (6). As the infection worsens, aquatic animals become inappetent, lethargic, and disoriented, and suffer necrotic subdermal cysts. Both the brown-spotted grouper and silvery black porgy exhibited an accumulation of pathogenic V. harveyi, with the spleens of these two species being the most favored organ for this pathogen, followed by the liver and kidneys (29).

In this study, the clinical symptoms of groupers infected with V. harveyi presented more diverse pathologies, including lethargy, inappetence, melanosis, irregular swimming behavior, superficial or deep ulcers on the head, and histological abnormalities in the hearts, gills, muscles, spleens, and livers. Similarly, in 2016, an aquaculture farm in Malaysia experienced an outbreak of V. harveyi-induced disease with an average daily mortality of 120 fish and total losses of 29% in just 10 days, and the infected fish exhibited lethargy, excessive mucus production, rotted fins, congestion of the liver and kidneys, and spleen enlargement (30). Similar situations have also occurred in many other warm regions, including Asia, southern Europe, and South America, in which the cultured marine fish and invertebrates experienced similar disease symptoms caused by pathogenic V. harveyi (6). However, none of these symptoms were detected in the fish living in cocultured *P. piscicida* and V. harveyi or in those living in monocultured *P. piscicida*, consistent with the fish not treated with these two strains (control group) in this study (Fig. 2), indicating that *P. piscicida* as an antagonistic bacterium could relieve or cure the symptoms caused by V. harveyi. We also monitored fish mortality daily for 10 days after challenge in the coculture and monocultures of *P. piscicida* and V. harveyi to evaluate the antagonistic effect of *P. piscicida* against V. harveyi and the safety of *P. piscicida* for groupers, and found that *P. piscicida* displayed a significantly protective effect on groupers against infection by V. harveyi (survival curve is shown in Fig. S3).

Generally, AKP and ACP have been shown to make significant contributions to the lysosome system of fish (31). SOD, a superoxide radical scavenging enzyme, was shown to protect tissue from injury caused by superfluous reactive oxygen products generated during the oxidative bactericidal process (32). MDA, a lipid-oxidation product, has been regarded as an important indicator of oxidative stress reflecting peroxidative tissue damage (33). All of these enzymes contribute greatly to the organism’s immune response. In this study, ACP, AKP, and SOD activity were remarkably higher and the MDA concentration significantly lower in all organs of groupers which were examined after V. harveyi infection alone compared to those examined after either *P. piscicida* infection alone or coculture infection; this strongly indicated an immune system response to the pathological invasion in which *P. piscicida*, as an inhibitor of V. harveyi, effectively protected the fish from triggering an immune response caused by V. harveyi.

All the *in vivo* results indicated that *P. piscicida* WCPW15003 was safe for aquatic animals, at least the pearl gentian grouper, and also suggested that *P. piscicida* WCPW15003 as an antagonistic bacterium could effectively protect aquatic animals from infection by pathogenic V. harveyi.

### Metabolomic and proteomic insight into the mechanism of *P. piscicida* activity against *V. harveyi*.

Given the harm caused by V. harveyi in mariculture water, many studies have aimed at controlling the spread of disease caused by this pathogen based on some excellent science, including the use of probiotics, one of the most promising methods for controlling V. harveyi infections without antibiotics. For example, Exiguobacterium acetylicum, a surface-associated bacterium, effectively prevented and controlled V. harveyi disease in brown-marbled grouper fingerlings (34), Vibrio lentus protected sea bass from challenge by V. harveyi (35), and Pseudoalteromonas flavipulchra JG1, a marine antagonistic bacterium, significantly inhibited the growth of V. harveyi (36). Many other probiotics against V. harveyi have been reported; however, most studies have focused on antagonistic and epigenetic features or biological phenomena instead of clarifying the antagonistic molecular mechanism.

It is well known that microbes generally do not exist in absolute isolation (22, 37, 38). We reason that it is crucial to explore the biological interplays of living microbes to elucidate the roles of biotic interactions in coexistence, such as antagonism, mutualism, commensalism, amensalism, and parasitism. Of these, antagonism based on Darwin’s competition-relatedness hypothesis is regarded as a basic piece of the puzzle of antagonistic molecular mechanisms in coexistence (39). Furthermore, microbes in coculture have been shown to influence the performance of antagonistic bacteria against pathogens by driving metabolic pathways during chemically mediated interspecies cross-talk (40). Additionally, antagonism as a defense strategy could be the driving force for discovering novel and powerful antibiotics which are absent in microbial monocultures. Indeed, coculture of species is undoubtedly complex compared to monoculture, and it is difficult to monitor the respective responses of both interacting microbes in terms of physiology and metabolism. Direct coculture allowing cell-to-cell interaction is one of the most realistic methods which mimic interspecies competition for precisely revealing functional molecular mechanisms. Therefore, it is essential to study pairwise or higher-order interactions of bacterial species in cocultures because all species, in combinations, may present mutualistic or antagonistic interplays by regulating metabolism and enzyme expression (39, 41).

Previous research reported that *Pseudoalteromonas* spp. preys on Gram-positive bacteria by producing pseudoalterin in a non-contact coculture (28); however, the information available on the antagonistic mechanism of *P. piscicida* against V. harveyi in contact coculture is limited. Given the complexity of the antagonistic process and the interactions between species in coculture, we performed a comprehensive multiomics analysis of the antagonistic *P. piscicida* and pathogenic V. harveyi in a coculture compared to a monoculture to provide new insights into the molecular mechanism of *P. piscicida* antagonism against V. harveyi. The untargeted metabolomics and proteomics methods used in this work produced an enormous array of metabolites and proteins participating in the specialized metabolisms of the coculture strains during the antagonistic process, which produced a highly useful assessment of global variation in the production of metabolites and proteins instead of focusing on specific compounds.

**FIG 9:**
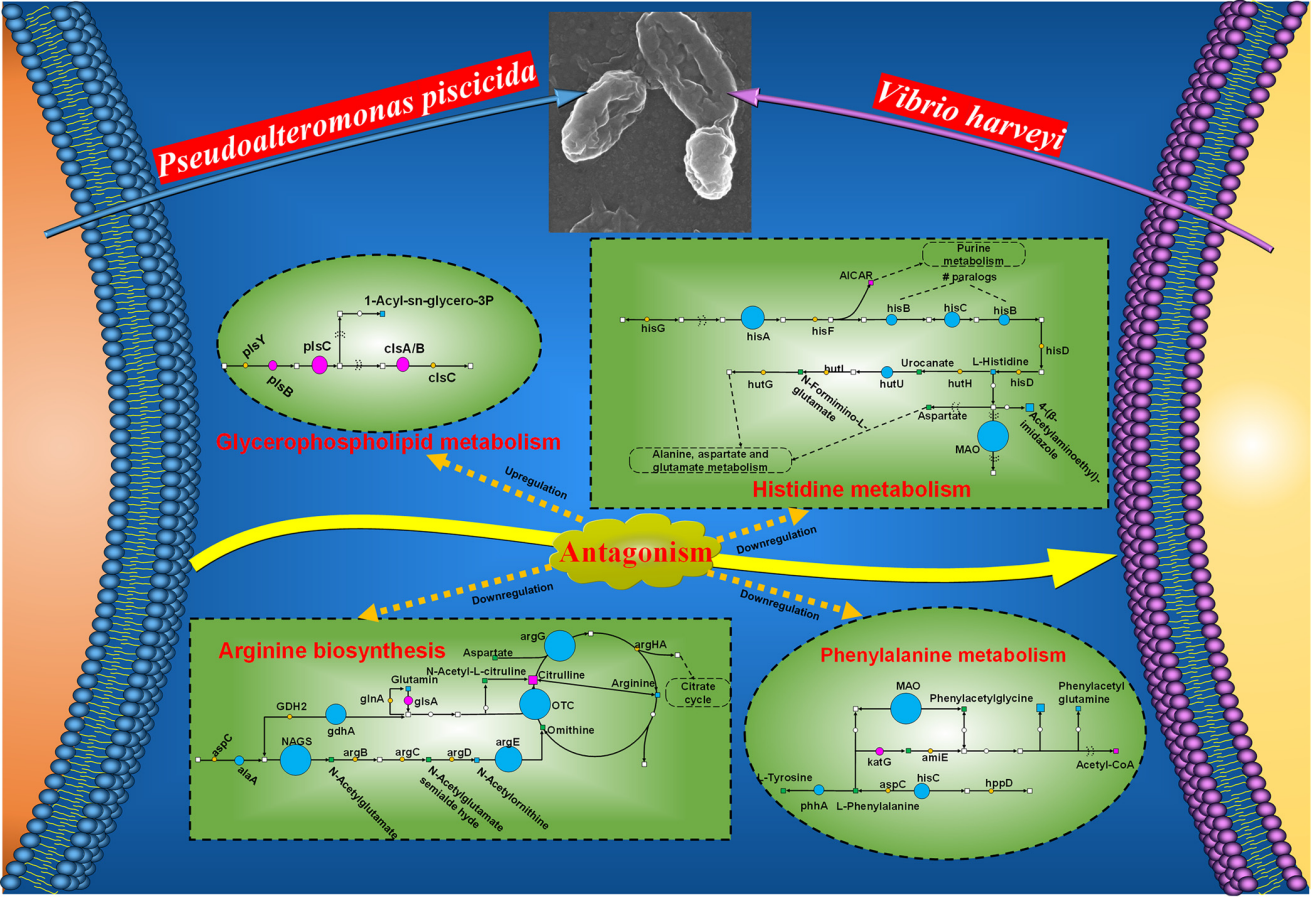
Proposed antagonistic mechanism of *P. piscicida* against V. harveyi in coculture.

During the process of antagonism in the coculture, we found that the antagonistic bacterium *P. piscicida* significantly downregulated the metabolic pathways of histidine metabolism, arginine biosynthesis, and phenylalanine metabolism, and upregulated the metabolic pathways of glycerophospholipid metabolism, leading to a competitive advantage against the co-occurring species V. harveyi. The metabolic pathways of histidine metabolism, phenylalanine metabolism, and arginine biosynthesis are usually found to be related to energy metabolism and pentose interconversions required by the growth of pathogenic microbes (42, 43); these findings were further supported by the antagonistic process of *P. piscicida* against V. harveyi, which showed dramatic downregulation in these three metabolic pathways. Additionally, the changes in these three metabolic pathways during the antagonistic process of *P. piscicida* against V. harveyi in coculture were similar to those of Acinetobacter johnsonii and Pseudomonas fluorescens (known as specific spoilage organisms) in coculture (22). *Pseudoalteromonas* spp. generally use specific physical properties of cellular membrane components (mainly glycerophospholipids) to modulate the membrane barriers against environmental stresses, which is usually regarded as a critical biological indicator (44, 45). In this study, glycerophospholipid metabolism was activated during the antagonistic process of *P. piscicida* against V. harveyi. In conclusion, we concluded that the costimulatory metabolic pathways of histidine metabolism, arginine biosynthesis, phenylalanine metabolism, and glycerophospholipid metabolism drove the antagonistic process of *P. piscicida* against V. harveyi (Fig. 9).

### Conclusion.

*P. piscicida* WCPW15003, a safe and effective antagonistic bacterium that specifically inhibits the dominant pathogenic V. harveyi in mariculture water, significantly and conclusively protected mariculture animals from V. harveyi infection by moderating the host immune response. Additionally, the antagonism of *P. piscicida* against V. harveyi was heavily driven by downregulation of the metabolic pathways of histidine metabolism, arginine biosynthesis, and phenylalanine metabolism and by the upregulation of glycerophospholipid metabolism, resulting in a competitive advantage of this probiotic against the dominant pathogenic V. harveyi in coculture. This work comprehensively elucidated the molecular mechanism of *P. piscicida* against the dominant pathogen in mariculture using modern molecular biology techniques and identified a promising probiotic agent for biocontrol of dominant pathogenic V. harveyi in mariculture water, providing a path toward a healthy and environmentally friendly recirculating-mariculture system.

## MATERIALS AND METHODS

### Bacterial strains and growth conditions.

P. piscicida WCPW15003 (CGMCC no. 23376; Fig. S1) was used to evaluate the antagonistic activity against pathogenic V. harveyi GDH11385 and their metabolomic and proteomic changes in coculture. These two strains were isolated from Hainan Island and stored in 30% glycerol solution at −80°C at the State Key Laboratory of Marine Resource Utilization in the South China Sea. In this study, *P. piscicida* and V. harveyi monocultures were defined as the P group and V group, respectively, the coculture was defined as the PV group, and a group without either bacterial species was the control group.

### Detection of antagonistic activity *in vitro*.

The antagonistic activity of *P. piscicida* against V. harveyi was assessed using a disk-diffusion assay. Briefly, 50-μL aliquots of V. harveyi broth culture (10 to 10^8^ CFU/mL) were plated on the surface of thiosulfate-citrate-bile salts-sucrose agar plates as the target plates. Twenty milliliters of *P. piscicida* bacterial culture were packed into a 50-mL centrifuge tube, then centrifuged at 10,000 × *g* for 10 min at room temperature to obtain the supernatant and bacterial cells of *P. piscicida* WCPW15003. Disks impregnated with different bacterial combinations of centrifuged supernatant and fermentations of *P. piscicida* (10 to 10^8^ CFU/mL) were placed on plates, and sterile 2166E seawater culture medium was used as the control. All plates were then incubated at 30°C. The mean clearing diameter (Δ*d*), which determined the antagonistic activity, was calculated from the difference between the diameter of the clearing zones of the target strains and the *P. piscicida* colony (bacterial cell)-containing discs (6.5 mm).

Additionally, 50-μL aliquots of broth cultures of *P. piscicida*, V. harveyi, and *P. piscicida* + V. harveyi with three biological replicates were incubated at 30°C in 48-well flat-bottomed plastic tissue culture plates filled with 950 μL sterile 2166E medium. The optical density (OD) of each well was measured at 600 nm at 1-h intervals in a microplate reader (BioTek Epoch 2 Microplate Spectrophotometer; Agilent Technologies, Winooski, VT, USA).

The antagonistic activity of *P. piscicida* against V. harveyi was investigated at the 0, 3, 9, and 12 h coculture stages using SEM. Briefly, 10 μL of coculture broth was spread on sterile glass coverslips, which were immersed in 2.5% (vol/vol) glutaraldehyde for fixation after drying in ambient air. The fixed coverslips were gradually dehydrated by ethanol (ranging from 50% to 100% with a gradient of 10%) for 10 min, dried in the ambient air, and subsequently sputter-coated with gold and viewed using SEM (Verios G4 UC; Thermo Fisher Scientific, Waltham, MA, USA).

### Detection of antagonistic activity *in vivo*.

**(i) Acclimatization of fish and management.** Healthy pearl gentian groupers (♀ *Epinephelus fuscoguttatus* × ♂ *Epinephelus lanceolatus*) with similar body weights of 33 ± 5 g were purchased from Hainan Lantaibang Biotechnology Co., Ltd. (Wenchang, Hainan, China), and divided into four groups (20 groupers per group) for the infection studies: control, P, V, and PV groups. The groupers were grown in 500-L containers with constant aeration and a temperature of 28 ± 2°C. The treatment groups (P, V, and PV groups) were immersed in a suspension with an initial bacterial concentration of about 10^5^ CFU mL^−1^ rearing water, while the control group was immersed in rearing water without any additional bacteria. Fish were fed once a day with formulated pellets provided by Hainan Lantaibang Biotechnology Co., Ltd. Pearl gentian groupers were acclimated to the indoor laboratory conditions for 1 week before the experiment. The entire *in vivo* experiment lasted 10 days. Groupers were anesthetized with tricaine methane sulfonate before tissue sample collection. Animal experiments complied with ethical standards and were approved by the Hainan University Institutional Animal Use and Care Committee (no. HNUAUCC-2021-00120).

**(ii) Histological examination and nonspecific immunological measurements.** After 10 days of rearing, the safety and antagonistic activity of *P. piscicida* against V. harveyi *in vivo* was evaluated by collecting the hearts, gills, muscles, spleens, and livers of groupers from the control, P, V, and PV groups. Tissue samples were divided into two parts and used for both the histological examination and to determine activities of immune-related enzymes. For the histological examination, one part of the tissues of the heart, gills, muscles, spleen, and liver was fixed in Davidson’s fixative (Shanghai Tarui Bioscience, Shanghai, China) for 24 h and dehydrated by a series of ethanol concentrations ranging from 50% to 100%, with a gradient of 50% → 70% → 80% → 90% → 95% → 100%. After dehydration, the tissues were equilibrated with xylene and embedded in paraffin. The paraffin blocks were cut into 5-μm thick slices, stained with hematoxylin and eosin, and examined for pathological alterations with an optical microscope.

For the nonspecific immunological measurements, the remaining tissue sections from the heart, gills, muscles, spleen, and liver were assayed for the immune-related enzymes acid-phosphatase (ACP), alkaline-phosphatase (AKP), malondialdehyde (MDA), and superoxide-dismutase (SOD) using commercial kits manufactured by Jiancheng Bioengineering Institute (Nanjing, China).

### Sample preparation for metabolomics and proteomics.

The P group, V group, and the coculture of equal amounts of these two strains (PV group) were cultured individually at 30°C in 2216E broth with shaking at 180 rpm for 24 h and were used for the metabolomic and proteomic analyses.

For the metabolomic analyses, all samples from the three groups with six replicates per sample were centrifuged at 12,000 × *g* for 5 min and then repeatedly washed with phosphate-buffered saline to isolate the cells. In addition, 400 μL 75% MeOH with 0.02 mg/mL 2-chloro*-*l*-*phenylalanine as an internal standard was added to 50 mg of each sample. The cells were treated with a high-throughput tissue crusher (−10°C, 50 Hz; Wonbio-96c; Shanghai Wonbio Technology Co., Ltd., Shanghai, China) to disrupt the cells and obtain the cell solution, which was used to extract intracellular metabolites using a low-temperature ultrasonic extraction method (5°C, 40 kHz; SBL-10TD; Ningbo Xinzhi Biotechnology Co., Ltd., Ningbo, China). After centrifugation for 10 min at 13,000 × *g*, the supernatant was used for further metabolic analysis. A mixture of aliquots from each supernatant sample was used as the quality control sample and periodically analyzed to monitor the stability of the instrument.

For the proteomic analyses, all samples from three groups with the three replicates per sample were dissolved 4-fold in lysis buffer containing 8 M urea and 1% protease inhibitors (Merck Millipore, Billerica, MA, USA). After ultrasonication, the total protein in the supernatant was obtained by centrifugation at 12,000 × *g* for 10 min at 4°C. Protein concentration in each sample was determined using a bicinchoninic acid (BCA) kit (Biyuntian Biotech Co., Ltd., Shanghai, China). All samples contained equal amounts of protein and were adjusted to equal volumes using lysis buffer. Next, 20% trichloroacetic acid (Sigma-Aldrich) was gradually added at 4°C for 2 h to obtain a precipitate. After repeated washings with precooled acetone, the precipitate was dried at room temperature, redissolved in N,N,N′,N′-tetramethyl-ethylenediamine to a final concentration of 200 mM, and hydrolyzed using 1/50 trypsin (m/m; Promega, Madison, WI, USA) for 24 h. Finally, 5 mM dithiothreitol was added to reduce the hydrolysate at 56°C for 30 min, and the reduced proteins were incubated with 11 mM iodoacetamide for 15 min in the dark.

### Analysis of metabolomics and proteomics.

**(i) Metabolomic analysis by UHPLC-QE/MS.** A total of 18 bacterial samples from the P, V, and PV groups were collected for a nontargeted metabolomics analysis by ultra-high-performance liquid chromatography coupled with Q Exactive Orbitrap mass spectrometry (UHPLC-QE/MS; Vanquish Horizon system; Thermo Fisher Scientific, Bremen, Germany), and the metabolites from each sample were separated by an ACQUITY UPLC HSS T3 column (2.1 mm × 100 mm × 1.8 m; Waters Corporation, Milford, MA, USA). The chromatography parameters for each sample are as follows. The column temperature was set at 40°C. The flow rate was 0.4 to 0.6 mL/min, and the sample injection volume was 2 μL. The mobile phases were prepared with water containing 5% acetonitrile and 0.1% formic acid as solvent A and 47.5% acetonitrile and 47.5% isopropanol containing 0.1% formic acid as solvent B. Additionally, the gradient program employed for the mobile phase was as follows: 0 to 3.5 min with a flow rate of 0.4 mL/min of 100% A; 3.5 to 5 min, flow rate of 0.4 mL/min of 75.5% A and 24.5% B; 5 to 5.5 min, flow rate of 0.4 mL/min of 35% A and 65% B; 5.5 to 7.4 min, flow rate of 0.4 mL/min of 100% B; 7.4 to 7.6 min, flow rate of 0.6 mL/min of 100% B; 7.6 to 7.8 min, flow rate of 0.6 mL/min of 48.5% A and 51.5 B; 7.8 to 9 min, flow rate of 0.5 mL/min of 100% A; 9 to 10 min, flow rate of 0.4 mL/min of 100% A; and 10 to 15 min, flow rate of 0.4 mL/min of 100% A. Mass spectrometry was carried out in both ESI (+) and ESI (−) mode: 70 to 1,050 (*m/z* 70 to 1,000) as the scan type; sheath gas flow rate, 50 arbitrary units (arb); auxiliary gas flow rate, 13 arb; heater temperature, 425°C; capillary temperature, 325°C; spray voltage, +3.5 kV for ESI (+) mode and −3.5 kV for ESI (−) mode; S-lens RF level, 55; resolution, 60,000 for full-scan MS and 7,500 for MS^2^; and MS/MS collision energies, 20, 40, and 60 eV.

**(ii) Proteomic analysis using nanoElute-UHPLC-trapped ion mobility spectrometry-time of flight (TIMS-TOF) MS.** The peptides from the P, V, and PV groups were dissolved in mobile phase A, then separated using the nanoElute UHPLC system (Bruker Daltonics, Bremen, Germany) with a Bruker Daltonics C_18_ column (1.9 μm, 75 μm × 100 mm). The chromatography gradient mobile phases were prepared with water containing 2% acetonitrile and 0.1% formic acid as solvent A and acetonitrile containing 0.1% formic acid as solvent B, and the flow rate was set to 450 nL/min. The gradient program employed for the mobile phase was as follows: 0 to 43 min with a linear increase in B from 6% to 22%; 43 to 55 min with a linear increase in B from 22% to 30%; 55 to 57 min with a linear increase in B from 30% to 80%; and 57 to 60 min in 80% B. The separated peptides were ionized by a capillary ion source at 1.75 kV. Next, the peptide parent ions and their secondary fragments were further analyzed using a timsTOF Pro mass spectrometer (Bruker Daltonics). The secondary mass spectrometry scan was operated in the range of 400 to 1,500 *m/z*. Additionally, the acquisition of mass spectra was collected using parallel accumulation-serial fragmentation (PASEF) mode, followed by 10 secondary spectral acquisitions in PASEF mode with parent ion charge numbers of 0 to 5. The MS/MS scan was repeated for the eight most abundant ions detected in the MS scan with dynamic exclusion set to 45 s.

### Mapping metabolic pathways.

Changes in metabolites and proteins were superimposed onto metabolic pathway maps obtained from Metabolon (http://www.metabolon.com) and the KEGG maps as modified. The metabolic pathways with metabolites and proteins detected with high confidence and which were significantly (fold change ≥ 1.5, *P* < 0.05) or very significantly changed (fold change ≥ 2, *P* < 0.05) were selected to reveal the differences in the metabolic composition and pathways between the PV and V groups and between the PV and P groups.

### Statistical analysis.

Metabolite data from UHPLC-QE/MS was imported into Progenesis QI software (Waters Corporation, Milford, MA, USA) for nonlinear retention time correction, peak filtration, extraction, and matching. The metabolite molecules and metabolic pathways were matched using the following databases: HMDB (http://www.hmdb.ca/), METLIN (http://metlin.scripps.edu/), iPath 3.0 (http://pathways.embl.de), and KEGG PATHWAY (http://www.genome.jp/kegg/). The protein data obtained from MS/MS were analyzed by MaxQuant (v1.6.15.0) with a false discovery of rate of <1%. Tandem mass spectra were searched against the protein database constructed from our genomes of *P. piscicida* (4,742 sequences) and V. harveyi (5,670 sequences). Trypsin/P was used as a cleavage enzyme, allowing up to two missing cleavages. The minimum peptide length and maximum number of peptide modifications were set to seven and five amino acid residues, respectively. The mass error tolerance of the secondary fragment ion was set to 20 ppm for precursor ions and fragment ions. Carbamidomethyl on Cys was set as a fixed modification, and oxidation on methionine and acetylation of the N termini of proteins were specified as variable modifications. The peptide ion score was set to ≥20 to improve = reliability. The false-positive rate was set to 1% for the accuracy of identification at the three levels of spectrum, peptide, and protein. Quantitative analysis was performed using intensity-based absolute quantification values.

A Student’s *t* test was used to identify statistically significant changes in metabolites and proteins (*P* < 0.05). Fold changes were calculated relatively with two comparison groups (PV compared to P or V group). Metabolite and protein comparisons with an FC of >1.5 (or <0.83) and a *t* test *P* value of <0.05 were considered to be differentially expressed. Additionally, the KEGG database was used to identify enriched pathways using a two-tailed Fisher’s exact test to test the enrichment of the differentially expressed proteins against all identified proteins. Corrections for multiple hypothesis testing were carried out using standard false discovery rate control methods. Pathways with a corrected *P* value of <0.05 were considered significant. These were classified into hierarchical categories according to the KEGG website. Statistical analyses of other experimental results were performed using SPSS v17.0 for Windows (SPSS Inc., Chicago, IL, USA), with *P* < 0.05 considered statistically significant.

### Data availability.

The genome of *P. piscicida* WCPW15003 has been deposited in the NCBI database under accession numbers CP081860 and CP081861.

## References

[1] Zhang W, Liang W, Li C. 2016. Inhibition of marine *Vibrio* sp. by pyoverdine from *Pseudomonas aeruginosa* PA1. J Hazard Mater 302:217–224. doi:10.1016/j.jhazmat.2015.10.003. 26476308

[2] Xu X, Liu K, Wang S, Guo W, Xie Z, Zhou Y. 2017. Identification of pathogenicity, investigation of virulent gene distribution and development of a virulent strain-specific detection PCR method for *Vibrio harveyi* isolated from Hainan Province and Guangdong Province, China. Aquaculture 468:226–234. doi:10.1016/j.aquaculture.2016.10.015.

[3] Yang A, Li W, Tao Z, Ye H, Xu Z, Li Y, Gao Y, Yan X. 2021. *Vibrio harveyi* isolated from marine aquaculture species in eastern China and virulence to the large yellow croaker (*Larimichthys crocea*). J Appl Microbiol 131:1710–1721. doi:10.1111/jam.15070. 33713523

[4] Lavilla-Pitogo CR, Baticados MCL, Cruz-Lacierda ER, Pena LDDL. 1990. Occurrence of luminous bacterial disease of *Penaeus monodon* larvae in the Philippines. Aquaculture 91:1–13. doi:10.1016/0044-8486(90)90173-K.

[5] Zhu Z, Duan C, Dong C, Weng S, He J. 2020. Epidemiological situation and phylogenetic relationship of *Vibrio harveyi* in marine-cultured fishes in China and Southeast Asia. Aquaculture 529:735652. doi:10.1016/j.aquaculture.2020.735652.

[6] Zhang X-H, He X, Austin B. 2020. *Vibrio harveyi*: a serious pathogen of fish and invertebrates in mariculture. Mar Life Sci Technol 2:231–245. doi:10.1007/s42995-020-00037-z. 32419972 PMC7223180

[7] Ren W, Wu H, Guo C, Xue B, Long H, Zhang X, Cai X, Huang A, Xie Z. 2021. Multi-strain tropical *Bacillus* spp. as a potential probiotic biocontrol agent for large-scale enhancement of mariculture water quality. Front Microbiol 12:699378. doi:10.3389/fmicb.2021.699378. 34456887 PMC8385719

[8] Wei Y, Bu J, Long H, Zhang X, Cai X, Huang A, Ren W, Xie Z. 2021. Community structure of protease-producing bacteria cultivated from aquaculture systems: potential impact of a tropical environment. Front Microbiol 12:638129. doi:10.3389/fmicb.2021.638129. 33613508 PMC7889957

[9] Ren W, Xu X, Long H, Zhang X, Cai X, Huang A, Xie Z. 2021. Tropical cellulolytic bacteria: potential utilization of sugarcane bagasse as low-cost carbon source in aquaculture. Front Microbiol 12:745853. 34777293 10.3389/fmicb.2021.745853 PMC8586208

[10] Pesci EC, Milbank JB, Pearson JP, McKnight S, Kende AS, Greenberg EP, Iglewski BH. 1999. Quinolone signaling in the cell-to-cell communication system of *Pseudomonas aeruginosa*. Proc Natl Acad Sci USA 96:11229–11234. doi:10.1073/pnas.96.20.11229. 10500159 PMC18016

[11] Ni N, Chou H-T, Wang J, Li M, Lu C-D, Tai PC, Wang B. 2008. Identification of boronic acids as antagonists of bacterial quorum sensing in *Vibrio harveyi*. Biochem Biophys Res Commun 369:590–594. doi:10.1016/j.bbrc.2008.02.061. 18295599

[12] Vaseeharan B, Ramasamy P. 2003. Control of pathogenic *Vibrio* spp. by *Bacillus subtilis* BT23, a possible probiotic treatment for black tiger shrimp *Penaeus monodon*. Lett Appl Microbiol 36:83–87. doi:10.1046/j.1472-765x.2003.01255.x. 12535126

[13] Wei Y, Wang F, Gao J, Huang Y, Ren W, Sheng H. 2021. Culture-dependent and culture-independent characterization of bacterial community diversity in different types of sandy lands: the case of Minqin County, China. BMC Microbiol 21:87. doi:10.1186/s12866-021-02150-0. 33752616 PMC7986352

[14] Pai SS, Anas A, Jayaprakash NS, Priyaja P, Sreelakshmi B, Preetha R, Philip R, Mohandas A, Singh ISB. 2009. *Penaeus monodon* larvae can be protected from *Vibrio harveyi* infection by pre-emptive treatment of a rearing system with antagonistic or non-antagonistic bacterial probiotics. Aquacult Res 41:847–860. doi:10.1111/j.1365-2109.2009.02362.x.

[15] Preetha R, Jose S, Prathapan S, Vijayan KK, Jayaprakash NS, Philip R, Singh ISB. 2010. An inhibitory compound produced by *Pseudomonas* with effectiveness on *Vibrio harveyi*. Aquacult Res 41:1452–1461. doi:10.1111/j.1365-2109.2009.02436.x.

[16] Rattanachuay P, Kantachote D, Tantirungkij M, Nitoda T, Kanzaki H. 2010. Inhibition of shrimp pathogenic vibrios by extracellular compounds from a proteolytic bacterium *Pseudomonas* sp W3. Electron J Biotechnol 13:8–9. doi:10.2225/vol13-issue1-fulltext-2.

[17] Ma C-W, Cho Y-S, Oh K-H. 2009. Removal of pathogenic bacteria and nitrogens by *Lactobacillus* spp. JK-8 and JK-11. Aquaculture 287:266–270. doi:10.1016/j.aquaculture.2008.10.061.

[18] Truong-Giang H, Hu S-Y, Chiu C-S, Truong Q-P, Liu C-H. 2019. Bacterial population in intestines of white shrimp, *Litopenaeus vannamei* fed a synbiotic containing *Lactobacillus plantarum* and galactooligosaccharide. Aquac Res 50:807–817. doi:10.1111/are.13951.

[19] Yan X, Gu S, Cui X, Shi Y, Wen S, Chen H, Ge J. 2019. Antimicrobial, anti-adhesive and anti-biofilm potential of biosurfactants isolated from *Pediococcus acidilactici* and *Lactobacillus plantarum* against *Staphylococcus aureus* CMCC26003. Microb Pathog 127:12–20. doi:10.1016/j.micpath.2018.11.039. 30496836

[20] Adel M, Yeganeh S, Dawood MAO, Safari R, Radhakrishnan S. 2017. Effects of *Pediococcus pentosaceus* supplementation on growth performance, intestinal microflora and disease resistance of white shrimp, *Litopenaeus vannamei*. Aquacult Nutr 23:1401–1409. doi:10.1111/anu.12515.

[21] Nakayama T, Lu H, Nomura N. 2009. Inhibitory effects of *Bacillus* probionts on growth and toxin production of *Vibrio harveyi* pathogens of shrimp. Lett Appl Microbiol 49:679–684. doi:10.1111/j.1472-765X.2009.02725.x. 19874484

[22] Wang X-Y, Xie J. 2020. Assessment of metabolic changes in *Acinetobacter johnsonii* and *Pseudomonas fluorescens* co-culture from bigeye tuna (*Thunnus obesus*) spoilage by ultra-high-performance liquid chromatography-tandem mass spectrometry. LWT Food Sci Technol 123:109073. doi:10.1016/j.lwt.2020.109073.

[23] Long RA, Azam F. 2001. Antagonistic interactions among marine pelagic bacteria. Appl Environ Microbiol 67:4975–4983. doi:10.1128/AEM.67.11.4975-4983.2001. 11679315 PMC93260

[24] Aiyar P, Schaeme D, Garcia-Altares M, Flores DC, Dathe H, Hertweck C, Sasso S, Mittag M. 2017. Antagonistic bacteria disrupt calcium homeostasis and immobilize algal cells. Nat Commun 8:1756. doi:10.1038/s41467-017-01547-8. 29170415 PMC5701020

[25] Akhter N, Wu B, Memon AM, Mohsin M. 2015. Probiotics and prebiotics associated with aquaculture: a review. Fish Shellfish Immunol 45:733–741. doi:10.1016/j.fsi.2015.05.038. 26044743

[26] Bhaskar N, Sudeepa E, Rashmi H, Selvi AT. 2007. Partial purification and characterization of protease of *Bacillus proteolyticus* CFR3001 isolated from fish processing waste and its antibacterial activities. Bioresour Technol 98:2758–2764. doi:10.1016/j.biortech.2006.09.033. 17092708

[27] Richards GP, Watson MA, Needleman DS, Uknalis J, Boyd EF, Fay JP. 2017. Mechanisms for *Pseudoalteromonas piscicida*-induced killing of vibrios and other bacterial pathogens. Appl Environ Microbiol 83:e00175-17. doi:10.1128/AEM.00175-17. 28363962 PMC5440704

[28] Tang B-L, Yang J, Chen X-L, Wang P, Zhao H-L, Su H-N, Li C-Y, Yu Y, Zhong S, Wang L, Lidbury I, Ding H, Wang M, McMinn A, Zhang X-Y, Chen Y, Zhang Y-Z. 2020. A predator-prey interaction between a marine *Pseudoalteromonas* sp. and Gram-positive bacteria. Nat Commun 11:285. doi:10.1038/s41467-019-14133-x. 31941905 PMC6962226

[29] Saeed MO. 1995. Association of *Vibrio harveyi* with mortalities in cultured marine fish in Kuwait. Aquaculture 136:21–29. doi:10.1016/0044-8486(95)01045-9.

[30] Mohamad N, Roseli FAM, Azmai MNA, Saad MZ, Yasin ISM, Zulkiply NA, Nasruddin NS. 2019. Natural concurrent infection of *Vibrio harveyi* and *V. alginolyticus* in cultured hybrid groupers in Malaysia. J Aquat Anim Health 31:88–96. doi:10.1002/aah.10055. 30536485

[31] Li J, Wu Z-B, Zhang Z, Zha J-W, Qu S-Y, Qi X-Z, Wang G-X, Ling F. 2019. Effects of potential probiotic *Bacillus velezensis* K2 on growth, immunity and resistance to *Vibrio harveyi* infection of hybrid grouper (*Epinephelus lanceolatus* male x *E. fuscoguttatus* female). Fish Shellfish Immunol 93:1047–1055. doi:10.1016/j.fsi.2019.08.047. 31425831

[32] Jiang J, Zhou Z, Dong Y, Jiang B, Chen Z, Yang A, Wang B, Guan X, Gao S, Sun H. 2016. The *in vitro* effects of divalent metal ions on the activities of immune-related enzymes in coelomic fluid from the sea cucumber *Apostichopus japonicus*. Aquac Res 47:1269–1276. doi:10.1111/are.12586.

[33] Yang G, Shen K, Yu R, Wu Q, Yan Q, Chen W, Ding L, Kumar V, Wen C, Peng M. 2020. Probiotic (*Bacillus cereus*) enhanced growth of Pengze crucian carp concurrent with modulating the antioxidant defense response and exerting beneficial impacts on inflammatory response via Nrf2 activation. Aquaculture 529:735691. doi:10.1016/j.aquaculture.2020.735691.

[34] Alipiah NM, Ramli NHS, Low C-F, Shamsudin MN, Yusoff FM. 2016. Protective effects of sea cucumber surface-associated bacteria against *Vibrio harveyi* in brown-marbled grouper fingerlings. Aquacult Environ Interact 8:147–155. doi:10.3354/aei00169.

[35] Schaeck M, Duchateau L, Van den Broeck W, Van Trappen S, De Vos P, Coulombet C, Boon N, Haesebrouck F, Decostere A. 2016. *Vibrio lentus* protects gnotobiotic sea bass (*Dicentrarchus labrax L.*) larvae against challenge with *Vibrio harveyi*. Vet Microbiol 185:41–48. doi:10.1016/j.vetmic.2016.01.024. 26931390

[36] Jin G, Wang S, Yu M, Yan S, Zhang X-H. 2010. Identification of a marine antagonistic strain JG1 and establishment of a polymerase chain reaction detection technique based on the *gyrB* gene. Aquacult Res 41:1867–1874. doi:10.1111/j.1365-2109.2010.02591.x.

[37] Chanos P, Mygind T. 2016. Co-culture-inducible bacteriocin production in lactic acid bacteria. Appl Microbiol Biotechnol 100:4297–4308. doi:10.1007/s00253-016-7486-8. 27037694

[38] Wei Y-L, Long Z-J, Ren M-X. 2022. Microbial community and functional prediction during the processing of salt production in a 1,000-year-old marine solar saltern of South China. Sci Total Environ 819:152014. doi:10.1016/j.scitotenv.2021.152014. 34852250

[39] Russel J, Roder HL, Madsen JS, Burmolle M, Sorensen SJ. 2017. Antagonism correlates with metabolic similarity in diverse bacteria. Proc Natl Acad Sci USA 114:10684–10688. doi:10.1073/pnas.1706016114. 28923945 PMC5635879

[40] Shi Y, Pan C, Cen S, Fu L, Cao X, Wang H, Wang K, Wu B. 2019. Comparative metabolomics reveals defence-related modification of citrinin by *Penicillium citrinum* within a synthetic *Penicillium-Pseudomonas* community. Environ Microbiol 21:496–510. doi:10.1111/1462-2920.14482. 30452116

[41] Niu B, Mu L, Xiao L, Zhang Z, Malakar PK, Liu H, Pan Y, Zhao Y. 2018. Reduction of infection risk mediated by co-culturing *Vibrio parahaemolyticus* and *Listeria monocytogenes* in refrigerated cooked shrimp. J Sci Food Agric 98:4454–4461. doi:10.1002/jsfa.8969. 29457648

[42] Liu Q, Wu J, Lim ZY, Lai S, Lee N, Yang H. 2018. Metabolite profiling of *Listeria innocua* for unravelling the inactivation mechanism of electrolysed water by nuclear magnetic resonance spectroscopy. Int J Food Microbiol 271:24–32. doi:10.1016/j.ijfoodmicro.2018.02.014. 29477806

[43] Kouremenos KA, Beale DJ, Antti H, Palombo EA. 2014. Liquid chromatography time of flight mass spectrometry based environmental metabolomics for the analysis of *Pseudomonas putida* bacteria in potable water. J Chromatogr B Analyt Technol Biomed Life Sci 966:179–186. doi:10.1016/j.jchromb.2014.02.058. 24674937

[44] Rühl J, Hein E-M, Hayen H, Schmid A, Blank LM. 2012. The glycerophospholipid inventory of *Pseudomonas putida* is conserved between strains and enables growth condition-related alterations. Microb Biotechnol 5:45–58. doi:10.1111/j.1751-7915.2011.00286.x. 21895997 PMC3815271

[45] Wang X, Xu Y, Song X, Jia Q, Zhang X, Qian Y, Qiu J. 2019. Analysis of glycerophospholipid metabolism after exposure to PCB153 in PC12 cells through targeted lipidomics by UHPLC-MS/MS. Ecotox Environ Safe 169:120–127. doi:10.1016/j.ecoenv.2018.11.006. 30445242

