# Supplementary material for: Multi-Costimulatory Pathways Drive the Antagonistic Pseudoalteromonas piscicida against the Dominant Pathogenic Vibrio harveyi in Mariculture: Insights from Proteomics and Metabolomics
Source: Microbiol Spectr. 2022 Oct 27;10(6):e02444-22. doi: 10.1128/spectrum.02444-22 (PMC9769913; doi:10.1128/spectrum.02444-22)
Supplement: Supplemental file 1 — Figure S1-S3 and Table S1-S4 legends. Download spectrum.02444-22-s0001.pdf, PDF file, 0.2 MB [file spectrum.02444-22-s0001.pdf]

Supplementary Material

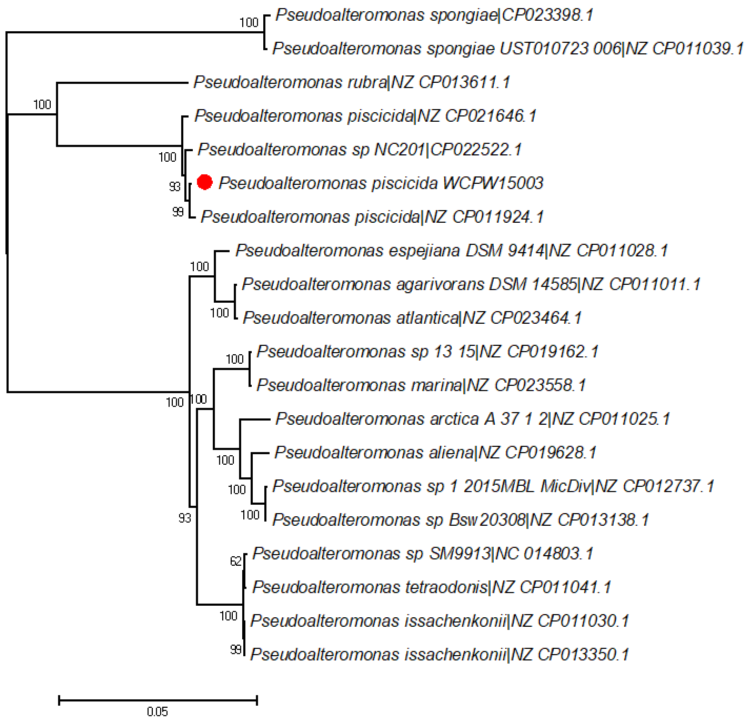

Figure S1 Phylogenetic tree of the housekeeping genes of *P. piscicida* WCPW15003

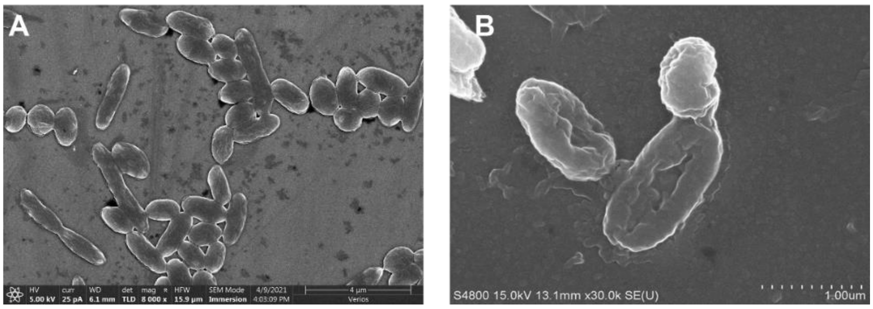

Figure S2 Close-up of the electron microscopy of *P. piscicida* and *V. harveyi* in co-culture.

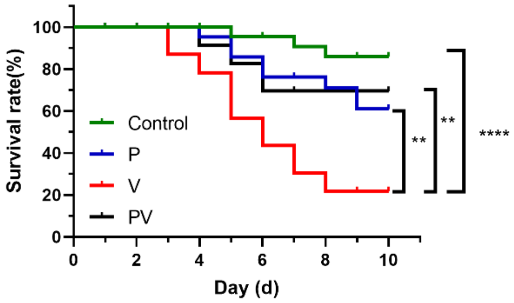

Figure S3 Survival rate of the control and infected groups after rearing for 10 days. Control group (A), P group (B), V group (C), and PV group (D).

Table S1 Overview of metabolomic variations during *P. piscicida* against *V. harveyi.*

Table S2 Overview of variations of proteomic during *P. piscicida* against *V. harveyi.*

Table S3 Differentially expressed metabolites and proteins in the PV compared to the P group.

Table S4 Differentially expressed metabolites and proteins in the PV compared to the V group.
